# Dichloroacetate and metformin synergistically suppress the growth of ovarian cancer cells

**DOI:** 10.18632/oncotarget.10694

**Published:** 2016-07-19

**Authors:** Bo Li, Xinzhe Li, Zhenhong Ni, Yan Zhang, Yijun Zeng, Xiaohuan Yan, Yan Huang, Jintao He, Xilin Lyu, Yaran Wu, Yuting Wang, Yingru Zheng, Fengtian He

**Affiliations:** ^1^ Department of Biochemistry and Molecular Biology, College of Basic Medical Sciences, Third Military Medical University, Chongqing 400038, China; ^2^ Department of Obstetrics and Gynecology, Daping Hospital and Research Institute of Surgery, Third Military Medical University, Chongqing 400042, China; ^3^ Cancer Center, Daping Hospital and Research Institute of Surgery, Third Military Medical University, Chongqing 400042, China; ^4^ Battalion 17 of Students, College of Preventive Medicine, Third Military Medical University, Chongqing 400038, China

**Keywords:** dichloroacetate, metformin, Mcl-1, cancer metabolism, ovarian cancer

## Abstract

Both dichloroacetate (DCA) and metformin (Met) have shown promising antitumor efficacy by regulating cancer cell metabolism. However, the DCA-mediated protective autophagy and Met-induced lactate accumulation limit their tumor-killing potential respectively. So overcoming the corresponding shortages will improve their therapeutic effects. In the present study, we found that DCA and Met synergistically inhibited the growth and enhanced the apoptosis of ovarian cancer cells. Interestingly, we for the first time revealed that Met sensitized DCA via dramatically attenuating DCA-induced Mcl-1 protein and protective autophagy, while DCA sensitized Met through markedly alleviating Met-induced excessive lactate accumulation and glucose consumption. The *in vivo* experiments in nude mice also showed that DCA and Met synergistically suppressed the growth of xenograft ovarian tumors. These results may pave a way for developing novel strategies for the treatment of ovarian cancer based on the combined use of DCA and Met.

## INTRODUCTION

The mortality of ovarian cancer ranks top among several types of gynecological cancers. At present, platinum and taxol-based chemotherapies are still standard paradigm in addition to surgery, however, their side effects are severe and the chemoresistance has also emerged [1–2]. Therefore, it is urgent to explore novel strategies as alternatives of traditional chemotherapy. In recent years, the growing evidences have shown that cancer is a kind of metabolic abnormalities, which pushes it to the forefront by regulating cancer metabolism to inhibit tumor growth [3]. Targeting key metabolic pathways significantly kill numerous cancer cells including ovarian cancer cells [4–5]. Among various metabolic drugs, dichloroacetate (DCA) and metformin (Met) have shown charming prospects because of their positive functions in cancer therapy.

As a mitochondria-targeting agent, DCA can inhibit the activity of pyruvate dehydrogenase kinase (PDK) and subsequently increase the activity of pyruvate dehydrogenase (PDH), which promotes the flux of carbohydrates into mitochondria and thereby enhances aerobic oxidation of glucose. This effect reverses mitochondrial dysfunction and reactivates mitochondria-dependent apoptosis in several tumor cells [6–9]. Simultaneously, DCA inhibits glycolysis and reduces lactate accumulation, which destroys the acidified tumor microenvironment (The acidified microenvironment is generally in favor of tumor survival) [10]. Although DCA has shown promising prospect in fighting against cancers, it has been reported that DCA induces protective autophagy in colon cancer cells which in turn hinders its apoptotic capacity [11]. So far, it is still unclear whether there is any other apoptosis-associated resistant determinant when DCA refreshes mitochondrial apoptosis.

Met is a traditional drug of first-line therapy for type 2 diabetes. Recent years, increasing evidences indicate that Met can also reduce the risk of cancer in several epidemiological studies [12]. Met suppresses tumor growth through inducing cycle arrest, promoting apoptosis and suppressing autophagy [13–15]. Furthermore, Met can sensitize some chemotherapeutic drugs such as paclitaxel, erlotinib, etc. [16–17]. More arrestingly, the anti-tumor effect of Met is increasingly linked to the glucose metabolism of cancer [18]. Despite several advantages in clinical trials, Met is hampered for further application because it might lead to lactate accumulation [19]. It is of great interest whether this disadvantage could be overcome by combining other metabolic drugs to make Met more extensively used in chemotherapy.

Given their potential mutual compensatory effects, we sought to uncover whether DCA and Met can synergize each other to enhance cytotoxicity in ovarian cancer cells. In the present study, we demonstrated that DCA and Met could collaboratively induce apoptosis in ovarian cancer cells. Met sensitized DCA via dramatically attenuating DCA-induce Mcl-1 and protective autophagy, while DCA sensitized Met through markedly alleviated Met-induced excessive lactate accumulation and glucose consumption. The *in vivo* experiments in nude mice also showed that DCA and Met synergistically suppressed the growth of xenograft ovarian tumors. These results suggest that this therapeutic strategy may be a promising choice for future metabolism-based targeted cancer therapy.

## RESULTS

### DCA and Met synergistically induce apoptosis in ovarian cancer cells

**Figure 1:**
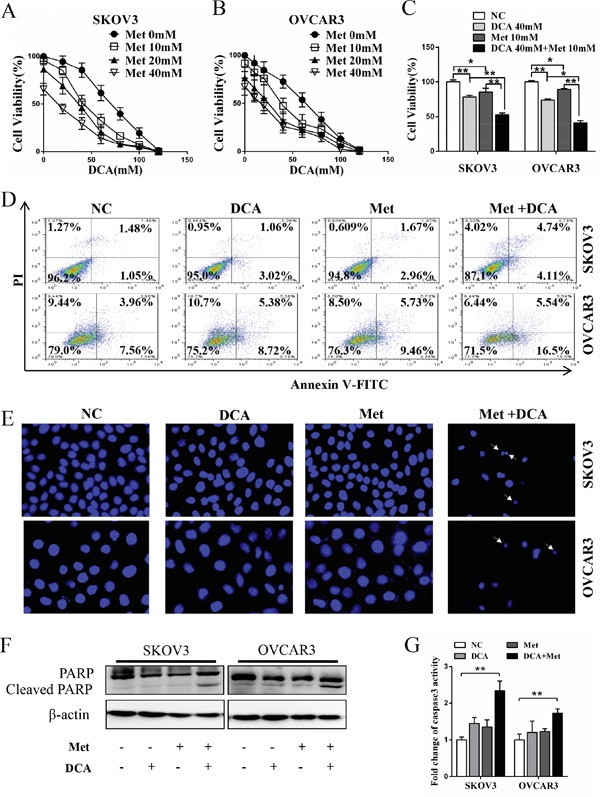
Dichloroacetate (DCA) and metformin (Met) synergistically induce apoptosis in ovarian cancer cells **A, B.** SKOV3 and OVCAR3 cells were treated with DCA and Met at indicated doses for 48 h, and then the cell viability was measured by CCK8 assay. **C.** SKOV3 and OVCAR3 cells were cotreated with 40 mM DCA and 10 mM Met or each alone for 48 h, and then the cell viability was measured by CCK8 assay. **D.** After treatment as in (C) for 24 h, the cells were stained with annexin V-FITC/PI. Then the percentage of apoptotic cells was calculated using flow cytometry. **E.** After treatment as in (D), the cell nucleus were stained with Hoechst 33258 and then observed under fluorescence microscope. The representative images were shown and the typical apoptotic bodies were marked with white arrows. **F, G.** After treatment as in (D), the cleavage of PARP was evaluated by Western blot (F), and the activation of caspase3 was measured by caspase3 activity assay (G). NC, negative control; *,*P*<0.05; **,*P*<0.01.

To investigate whether there is a synergistic effect between DCA and Met in suppressing the growth of ovarian cancer cells, SKOV3 and OVCAR3 cells were cotreated with DCA and Met or each alone. As shown in Figure 1A-1C, cotreatment with DCA and Met more efficiently repressed the growth of the ovarian cancer cells compared to each alone, and the combination of 40mM DCA and 10mM Met could inhibit the cell viability to as low as 50% compared to the control. So we chose 40mM DCA and 10mM Met in the subsequent experiments. Similarly, the synergistic inhibition effect was also observed in cervical cancer cells (HeLa and SiHa), non-small cell lung cancer cells (A549 and GLC-82) and human hepatocellular carcinoma cells (HepG2) (Figure S1A-1D), suggesting that the synergism between DCA and Met may be universal to some extent. Moreover, DCA and Met synergistically induced apoptosis in ovarian cancer cells revealed by Flow Cytometry analysis of annexin V-FITC (fluorescein isothiocyanate) and PI (prodium iodide) double staining (Figure 1D), Hoechst staining of apoptotic bodies (Figure 1E), Western blot analysis of cleaved PARP (poly ADP-ribose polymerase, a marker of apoptosis) (Figure 1F) and caspase3 activity assay (Figure 1G).

### Met sensitizes DCA via attenuating DCA-induced Mcl-1

**Figure 2:**
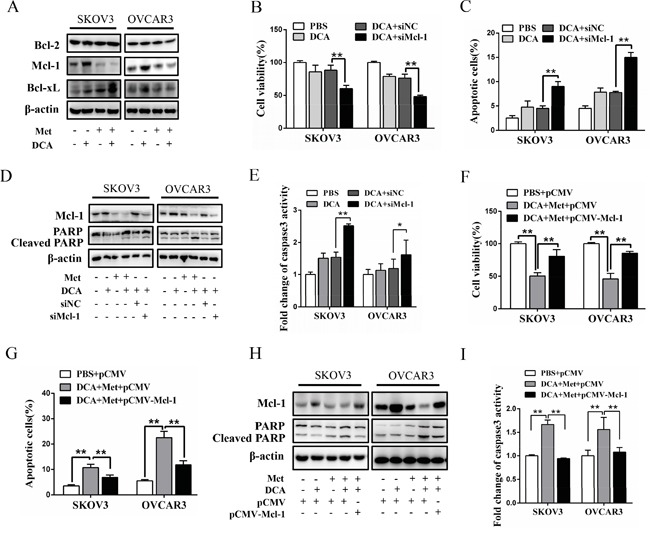
Met sensitizes DCA through decreasing DCA-induced Mcl-1 **A.** SKOV3 and OVCAR3 cells were cotreated with 40 mM DCA and 10 mM Met or each alone for 24 h, then Bcl-2, Bcl-xL and Mcl-1 were detected by Western blot. **B-E.** After transfection with Mcl-1 siRNA or control siRNA for 12 h, the cells were treated with 40 mM DCA or PBS control for another 24 h. Then the cell viability was detected using CCK8 assay (B), the percentage of apoptotic cells was calculated using flow cytometry (annexin V-FITC/PI) (C), the levels of Mcl-1 and cleaved PARP were examined with Western blot (D) and caspase3 activity was measured by caspase3 activity assay (E). **F-I.** After transfection with Mcl-1 expressing plasmid or control plasmid for 12 h, the cells were cotreated with 40 mM DCA and 10mM Met for another 24 h. Then cell viability (F), the percentage of apoptotic cells (G), the levels of Mcl-1 and cleaved PARP (H) and caspase3 activity (I) were assayed as in (B-E). siNC, siRNA for negative control; siMcl-1: siRNA for Mcl-1; *,*P*<0.05; **,*P*<0.01.

To explore the mechanism by which Met sensitizes DCA to induce apoptosis, we examined the expression of the crucial antiapoptotic Bcl-2 family members including Mcl-1, Bcl-2 and Bcl-xL [20]. As shown in Figure 2A, DCA alone significantly increased the level of Mcl-1 protein (but not Bcl-2 and Bcl-xL proteins) in ovarian cancer cells, which was markedly attenuated by Met. Silence of Mcl-1 by siRNA enhanced the DCA-mediated inhibition of the cell viability (Figure 2B), and augmented DCA-induced apoptosis (Figure 2C-2E). Moreover, ectopic expression of Mcl-1 dramatically alleviated the sensitizing effect of Met to DCA on cell viability and apoptosis (Figure 2F-2I). These results indicated that Mcl-1 is a novel resistant factor of DCA, and Met sensitizes DCA via downregulating Mcl-1.

### Met attenuates DCA-induced Mcl-1 through inhibiting Mcl-1 translation

**Figure 3:**
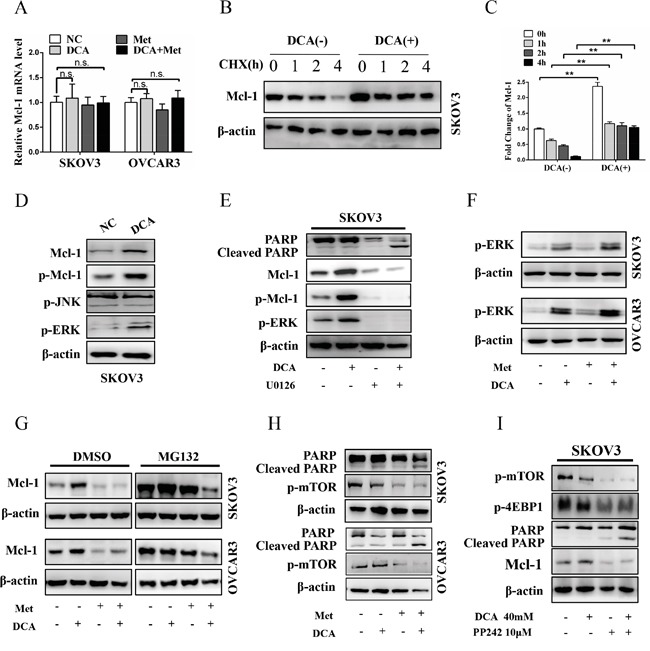
Met attenuates DCA-induced Mcl-1 through inhibiting Mcl-1 translation **A.** After cotreatment with 40 mM DCA and 10 mM Met or each alone for 24 h, the mRNA level of Mcl-1 was examined by qPCR, and the data was expressed as the fold change over the control. **B, C.** After treatment with 40 mM DCA or PBS control for 24 h, 100μM CHX (translational inhibitor) was added at indicated times before the cells were harvested. Then the expression of Mcl-1 was detected by Western blot (B). The intensity of the protein bands were quantified by Quantity One software from Bio-Rad Company, and the ratios of Mcl-1/β-actin were shown in (C). **D.** The cells were treated with 40 mM DCA or PBS for 24 h, and then the levels of p-ERK, p-JNK, p-Mcl-1 and total Mcl-1 were determined by Western Blot. **E.** After pretreated with 10μM U0126 (MEK1/2 inhibitor) or vehicle control DMSO for 2 h, the cells were treated with 40 mM DCA or PBS for another 24 h. Then the level of p-Mcl-1, p-ERK, total Mcl-1 and cleaved PARP were analyzed by Western blot. **F.** The cells were treated as in (A), and then the level of p-ERK was measured by Western blot. **G.** After treated as in (A) for 22 h, the cells were incubated with 10μM MG132 (proteasome inhibitor) or DMSO for another 2 h. Then the level of Mcl-1 protein was tested by Western blot. **H.** The cells were treated as in (A), and then the level of cleaved PAPR and p-mTOR were assessed by Western blot. **I.** After pretreated with DMSO or 2μM PP242 (mTOR inhibitor) for 2 h, the cells were treated with 40 mM DCA or PBS for another 24 h. Then the level of p-mTOR, p-4EBP1, Mcl-1 and cleaved PARP were analyzed by Western blot. n.s., no significance; **,*P*<0.01.

To elucidate in which level DCA induces Mcl-1, the mRNA of Mcl-1 was firstly examined. As shown in Figure 3A, DCA had no obvious effect on Mcl-1 mRNA expression. Subsequently, Mcl-1 protein was analyzed in the presence or absence of translational inhibitor cycloheximide (CHX). As shown in Figure 3B and 3C, CHX time-dependently decreased the basal (but not DCA-induced) Mcl-1 protein, indicating that DCA increases the stability of Mcl-1 protein. It has been reported that the phosphorylated ERK (p-ERK) and p-JNK can stabilize Mcl-1 through phosphorylating Mcl-1 on Thr^163^ [21–22], so we investigated whether p-ERK/p-JNK is involved in the regulation of DCA-induced Mcl-1. As shown in Figure 3D, treatment with DCA significantly elevated p-ERK (but not p-JNK) and p-Mcl-1^Thr163^ in ovarian cancer cells. Moreover, the MEK1/2 inhibitor U0126 could dramatically attenuated DCA-induced Mcl-1 and p-Mcl-1^Thr163^, and markedly strengthened the cleaved PARP (Figure 3E). These results indicated that p-ERK (but not p-JNK) is a mediator of DCA-induced Mcl-1. Previous studies have demonstrated that DCA elevates Reactive Oxygen Species (ROS) [23], and ROS is a key inducer of p-ERK [24], so we examined the level of ROS with DCFH-DA. As shown in Figure S2A, DCA increased the generation of ROS, suggesting that induction of ROS may be a mechanism by which DCA enhances p-ERK activation.

Additionally, Ser^159^ is also closely related with Mcl-1 stability and this site is mainly phosphorylated by GSK-3β [25], so we tested whether GSK-3β is also involved in the regulation of DCA-induced Mcl-1 stabilization. As shown in Figure S2B, DCA increased the phosphorylation of GSK-3β, but had no effect on total GSK-3β. Moreover, DCA enhanced the phosphorylation of Akt (an upstream signal molecule of GSK-3β), and Akt inhibitor MK-2206 2HCl dramatically attenuated DCA-induced GSK-3β phosphorylation, Mcl-1 upregulation and apoptosis resistance (Figure S2C). These results indicated that p-Akt-mediated phosphorylation of GSK-3β promotes the DCA-induced Mcl-1 stabilization.

Subsequently, we examined whether Met can attenuate DCA-induced Mcl-1 by inhibiting p-ERK/p-Akt. As shown in Figure 3F and Figure S2D, Met could not suppress the DCA-induced p-ERK and p-Akt, together with the results in Figure 3A, indicating that Met decreases DCA-induced Mcl-1 neither at transcriptional nor at post-translational level. Then we analyzed whether Met attenuates DCA-induced Mcl-1 at translational level with the proteasome inhibitor MG132. As shown in Figure 3G, when cells were treated with control, DCA, Met or combination, the protein levels of Mcl-1 were equally elevated in the presence of MG132 compared to DMSO, indicating that Met attenuates DCA-induced Mcl-1 via inhibiting Mcl-1 translation. It has been reported activation of mTOR promotes Mcl-1 translation [26], so we analyzed p-mTOR after cotreatment with Met and DCA. As expected, Met markedly decreased the level of p-mTOR (Figure 3H), and the mTOR inhibitor PP242 had the similar effect to Met on promoting apoptosis (Figure 3I). According to the data in Figure 3 and Figure S2, we can conclude that DCA upregulates Mcl-1 via enhancing the phosphorylation of ERK and GSK-3β, and Met suppresses Mcl-1 translation through inhibiting p-mTOR.

### Met diminishes DCA-induced protective autophagy

**Figure 4:**
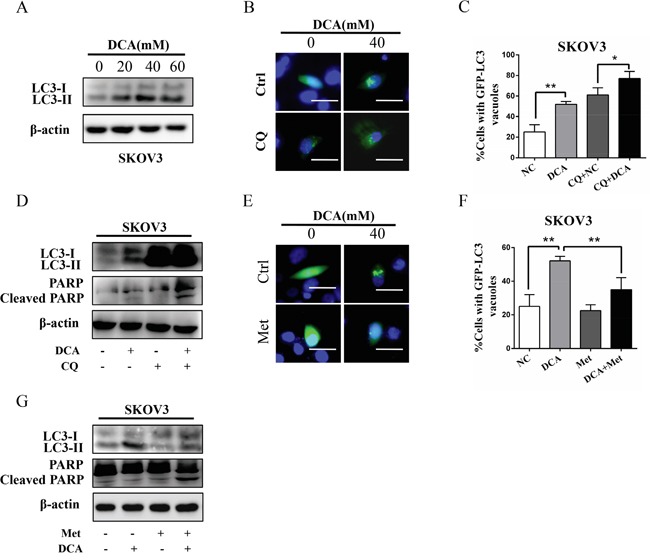
Met diminishes DCA-induced protective autophagy **A.** SKOV3 cells were treated with the indicated concentrations of DCA, and then the levels of LC3B-I/II were detected by Western blot. **B, C.** After transfected with GFP-LC3 expressing plasmid for 12 h, SKOV3 cells were treated with 40 mM DCA for another 24 h in the presence or absence of the autophagy inhibitor CQ (20 mM). Then the green fluorescent GFP-LC3 punctas (which occurred upon autophagy induction) were observed under fluorescent microscope (B). Subsequently, the data from (B) was quantified and expressed as the percentage of the cells containing 5 or more GFP-LC3 punctas (C). **D.** SKOV3 cells were treated with 40 mM DCA for 24 h in the presence or absence of the CQ (20 mM), and then the levels of LC3-I/II and cleaved PARP were examined by Western blot. **E, F.** After transfected with GFP-LC3 expressing plasmid for 12 h, SKOV3 cells were cotreated with 40 mM DCA and 10 mM Met or each alone for 24 h. Then the green fluorescent GFP-LC3 punctas were photographed (E) and quantified (F) as in (B and C). **G.** SKOV3 cells were cotreated with 40 mM DCA and 10 mM Met or each alone for 24 h, and then the levels of LC3-I/II and cleaved PARP were detected by Western blot. *,*P*<0.05; **,*P*<0.01.

Previous studies have shown that autophagy plays an important role in the therapeutic resistance of DCA in colon cancer cells [11], so we examined the role of autophagy in ovarian cancer cells upon treatment with DCA or/and Met. As shown in Figure 4A, DCA dose-dependently promoted the level of MAP1LC3-II (LC3-II), the marker of autophagy. Inhibition of autophagy by chloroquine (CQ) or silence of ATG7 dramatically enhanced the DCA-induced apoptosis and cytotoxicity (Figure 4B-4D, Figure S3A-3D), indicating that DCA induces protective autophagy in ovarian cancer cells. To preliminary investigate the mechanism of DCA-induced autophagy, we scanned the mRNA changes of 7 autophagy-related genes in DCA-treated cells. As shown in Figure S3E, DCA dramatically upregulated the mRNA level of ATG7 in ovarian cancer cells, suggesting that ATG7 may be involved in the DCA-induced protective autophagy. Subsequently, we found that Met remarkably decreased the DCA-induced LC3-II (Figure 4E-4G), indicating that Met could attenuate the DCA-induced protective autophagy. In summary, it could be drawn that weakening DCA-induced protective autophagy is also important in the sensitizing effect of Met to DCA.

### DCA alleviates Met-induced glucose consumption and lactate production

**Figure 5:**
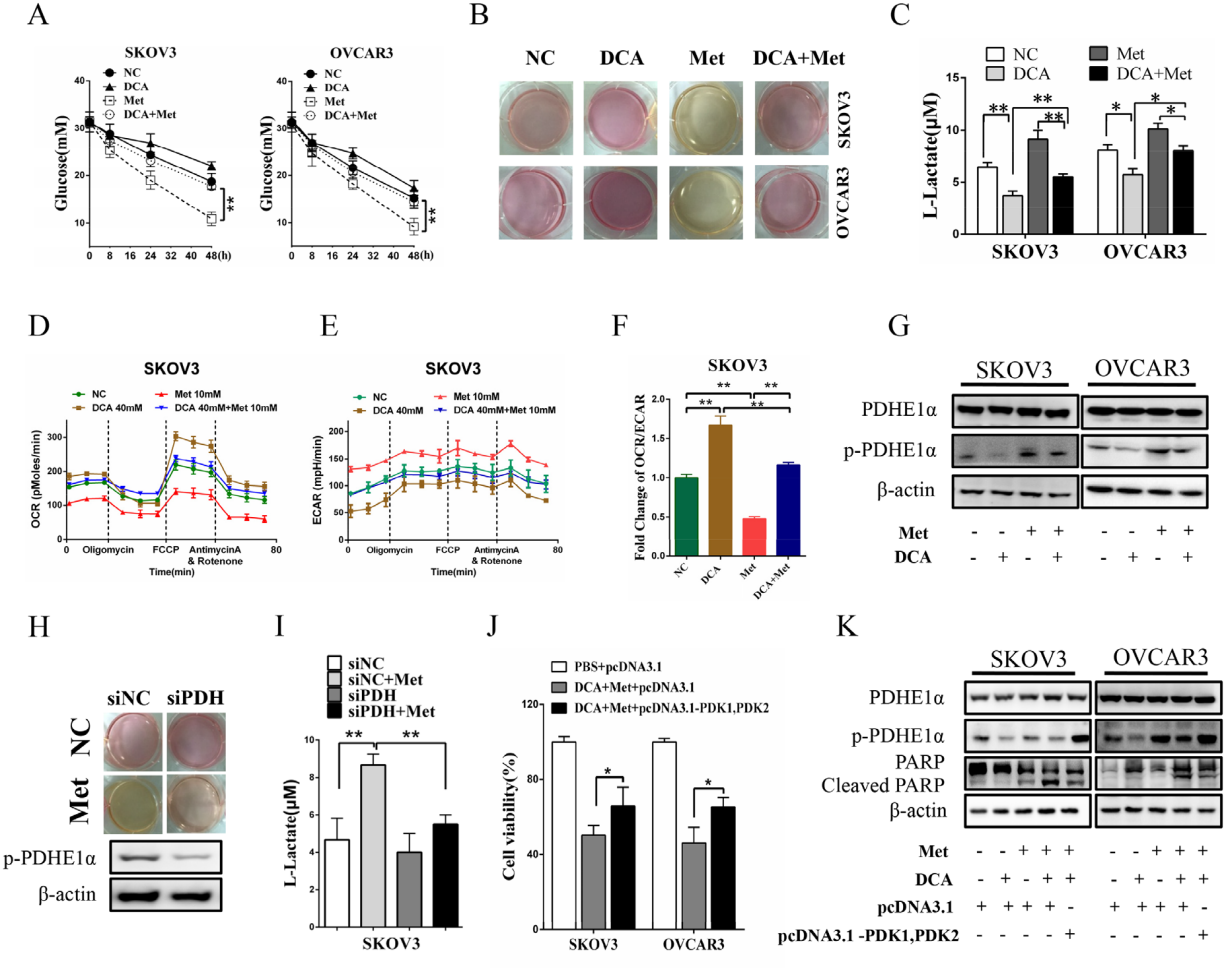
DCA alleviates Met-induced glucose consumption and lactate production **A.** SKOV3 and OVCAR3 cells were cotreated with 40 mM DCA and 10 mM Met or each alone for the indicated times, and then the concentrations of glucose in the culture media were measured separately. **B, C.** After cotreatment with 40 mM DCA and 10 mM Met or each alone for 24h, the color of the culture media were photographed (B), and the concentrations of L-lactate in the media were assayed (C). **D-F.** After treated as in (B), the OCR (D) and ECAR (E) of the cells were measured by XF Cell Mito Stress Test Kit, and the mitochondrial respiration rate (OCR/ECAR) was calculated (F). **G.** The cells were treated as in (B), and then the levels of total PDHE1α and p-PDHE1α on Ser^293^ were detected by Western blot. **H, I.** After transfected with the siRNA for PDH or control siRNA for 12h, SKOV3 cells were treated with 10mM Met or PBS. Then the color of the culture media were photographed (H), and the concentrations of L-lactate in the media were assayed (I). **J, K.** After cotransfected with PDK1 and PDK2 expression vectors (pcDNA3.1- PDK1, PDK2) or control vector pcDNA3.1 for 12 h, the cells were cotreated with 40 mM DCA and 10 mM Met for another 24 h. Then the cell viability was determined by CCK8 assay (J), and the levels of total PDHE1α, p-PDHE1α on Ser^293^ and cleaved PARP were analyzed by Western blot. siNC, siRNA for negative control; siPDH: siRNA for PDH; *,*P*<0.05; **,*P*<0.01.

To clarify whether DCA can overcome the shortages of Met, the changes of lactate and glucose were analyzed. As shown in Figure 5A-5C, Met increased lactate production and glucose consumption, which was remarkably alleviated by DCA. Moreover, the cellular oxygen consumption rate (OCR) and extracellular acidification rate (ECAR) were measured. As shown in Figure 5D-5F, Met decreased the ratio of OCR/ECAR, which was dramatically attenuated by DCA, revealing that DCA can suppress the Met-induced glycolysis via recovering mitochondrial respiration. As DCA is an inhibitor of PDKs which phosphorylates and inhibits the activity of PDH [9], so we examined the level of p-PDH. As shown in Figure 5G, Met elevated the level of p-PDHE1α (a subunit of PDH), which was markedly reversed by DCA. Silence of PDH with siRNA significantly attenuated the Met-induced lactate production (Figure 5H, 5I). Ectopic expression of PDK1 and PDK2 enhanced the phosphrylation of PDHE1α and attenuated apoptosis induced by the cotreatment with DCA and Met (Figure 5J, 5K), taken together with the data in Figures 5G-5I, indicating that DCA can sensitize Met through inhibiting PDK/PDH pathway in killing ovarian cancer cells.

### DCA and Met collaboratively repress the growth of ovarian cancer cells *in vivo*

**Figure 6:**
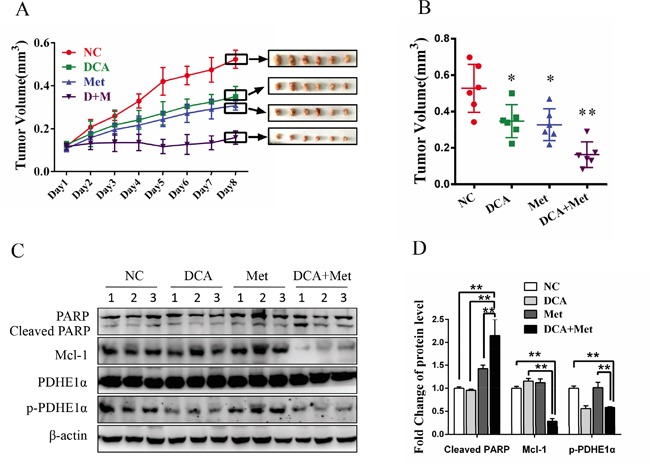
DCA and Met collaboratively repress the growth of ovarian cancer cells *in vivo* **A-D.** 5×10^6^ SKOV3 cells in 150 μL PBS were implanted into the right axillae of each nude mouse. When palpable tumors were formed, the mice were randomized into 4 groups (n = 6 per group). Then the mice were intraperitoneally injected everyday with DCA (50 mg/kg/d) plus Met (100 mg/kg/d) or each alone for 8 days, taking PBS as control. The xenograft tumor size was monitored every day (volume = width^2^×length×1/2) (A). After excised from the mice, the xenograft tumors were photographed (A) and their volumes were showed in (B). The levels of cleaved PARP, Mcl-1, total PDHE1α and p-PDHE1α were measured by Western blot (C), and the ratios of the corresponding proteins to β-actin were calculated (D). *,*P*<0.05; **,*P*<0.01.

As shown in Figure 6A and 6B, cotreatment with DCA and Met more efficiently suppressed the growth of ovarian cancer xenografts in nude mice compared to the treatment with DCA or Met alone. Western blot analysis showed that DCA and Met synergistically increased cleaved PARP, and downregulated Mcl-1 and p-PDHE1α in the xenografts (Figure 6C-6D). These results suggest that DCA and Met can collaboratively inhibit the growth of ovarian cancer cells *in vivo* through attenuating the shortages of each other.

## DISCUSSION

**Figure 7:**
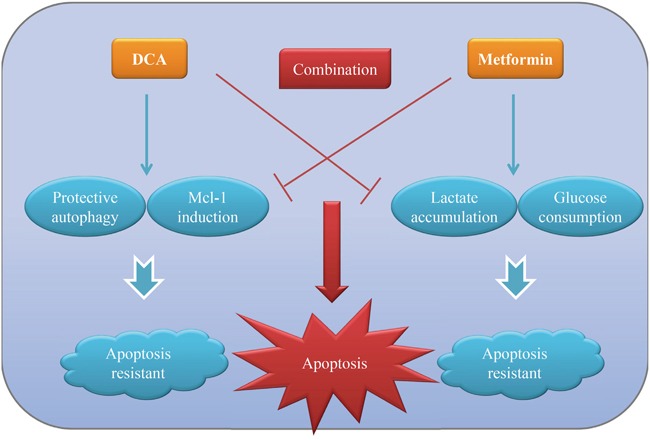
The working model for the synergistic sensitization of DCA and Met to each other in ovarian cancer cells DCA can induce anti-apoptotic protein Mcl-1 and protective autophagy, which in turn inhibits DCA-induced apoptosis in ovarian cancer cells. Met can result in lactate accumulation and high glucose consumption, which hampers it to kill ovarian cancer cells. DCA and Met can synergistically induce apoptosis of ovarian cancer cells via overcoming the reciprocal shortages.

It has been confirmed that most solid tumors are characterized by “Warburg effect” whereby they use glycolysis for energy production even though oxygen is sufficient. Targeting this abnormal phenomenon has paved a way for developing novel cancer therapeutic strategies in addition to traditional cytotoxic drugs. In the present study, we demonstrated that cotreatment with DCA and Met (two metabolic-associated agents) can more efficiently repress the growth of ovarian cancer cells compared to each alone *in vitro* and *in vivo*. Met attenuates DCA-induced Mcl-1 and protective autophagy, while DCA alleviates Met-induced excessive lactate accumulation and glucose consumption. The reciprocal benefits of the two agents contribute an intense apoptosis to kill ovarian cancer cells more effectively. The working model of DCA and Met in combination was shown in Figure 7.

Although studies have been undergoing for years, the key factors which may impede the pro-apoptotic effect of DCA are not yet clear. Our results showed that Mcl-1 is a crucial resistant factor against DCA-induced apoptosis in ovarian cancer cells, and cotreatment with Met and DCA decreased Mcl-1 and enhanced apoptosis. However, the cotreatment led to an increase of Bcl-xL (Figure 2A), which may be as a compensatory mechanism to keep the cell survival. The similar phenomenon has also been reported in previous study [27] (The study shows that Bcl-2/xL inhibitor ABT-263 induces cancer cell apoptosis while upregulates Mcl-1). Moreover, the cotreatment-induced Bcl-xL increase was only present in SKOV3 (but not in OVCAR3) cell line, suggesting that this effect may be cell specific. Of course, it remains to be determined about the detailed mechanism by which the cotreatment with Met and DCA upregulates Bcl-xL. By further study, we revealed that DCA induced Mcl-1 accumulation via activating ERK and Akt, which protected Mcl-1 from proteasome-mediated degradation. These findings suggest that inhibition of ERK and Akt may be a good strategy to sensitize DCA in ovarian cancer treatment. However, a conflict result has been reported recently that DCA decreases the level of Mcl-1 in AML cells [28] and colorectal cancer cells [29]. The discrepancies suggest that the relationship between DCA and Mcl-1 may be largely different in different contexts.

Autophagy is a catabolic process to recycle essential metabolites such as amino acids and lipids for replenishing their bioenergetic reserve in the presence of nutrients deprivation or other dramatic stresses [30–31]. In this study, we revealed that DCA induced a protective autophagy in ovarian cancer cells, and ATG7 may play a role in this process (Figure S3E), but the detailed mechanism needs to be further studied. Moreover, we found that Met sensitized DCA via suppressing the DCA-induced protective autophagy. Consistent with our findings, Met can repress GRP78-dependent autophagy to enhance the anti-myeloma effect of bortezomib [15], and inhibit 2DG-induced autophagy to sensitize 2DG in prostate cancer cells [32]. However, the detailed mechanism(s) by which Met suppresses the protective autophagy remains to be further investigated.

Met has been well recognized as a single agent or sensitizer in cancer therapy, but a notable disadvantage is that Met promotes glucose consumption and accelerates lactate accumulation which facilitates cancer-addicted aerobic glycolysis. In the present study, we demonstrated that DCA could dramatically attenuate this side effect. DCA can strongly inhibits the activity of PDKs and its downstream p-PDHE1α, leading to a metabolic remodeling reusing oxidative phosphorylation and causing lower lactate accumulation and glucose consumption. Among the four types of PDK isoenzymes, DCA mainly functions through inhibiting PDK2 and PDK1 [33]. As expected, concurrent over-expression of PDK2 and PDK1 enhanced the phosphorylation of PDHE1α, abolished the sensitizing function of DCA and partially abrogated the lethal effect of DCA plus Met in ovarian cancer cells. Taken together, our results indicate that DCA sensitizes the anti-tumor function of Met through inhibiting the activity of PDKs. However, it should be noted that DCA can sensitize Met by enhancing Met-induced oxidative stress in breast cancer cells [34]. This means the synergizing mechanism of DCA to Met is so complicated, which needs deep studies.

In summary, we showed that DCA and Met can synergistically suppress the growth of ovarian cancer cells, which may pave a way for developing novel strategies for the treatment of ovarian cancer based on the combined use of DCA and Met.

## MATERIALS AND METHODS

### Cell lines and reagents

The cell lines including SKOV3, OVCAR3, HeLa, SiHa, GLC-82, A549 and HepG2 were purchased from American Type Culture Collection (ATCC) and were cultured in Dulbecco's Modified Eagle Medium (DMEM), supplemented with 10% fetal bovine serum (FBS), streptomycin (100 mg/mL) and penicillin (100 U/mL) at 37°C in a 5% CO_2_ humid incubator. DCA and Cycloheximide (CHX) were purchased from Sigma-Aldrich (Louis, MO, USA). Met, U0126, MG132 and Hoechst 33258 were purchased from Beyotime Company (Shanghai, China). MK2206 was purchased from Selleck Company (Shanghai, China). Caspase-3 Activity Assay Kit and Reactive Oxygen Species Assay Kit were purchased from Beyotime Company (Shanghai, China). Annexin V-FITC and PI were purchased from BD Bioscience (BD, NJ, USA). siRNAs to Mcl-1, ATG7, PDH and control siRNA were from GenePharma (Shanghai, China). pCMV and pcDNA3.1, pCMV-Mcl-1, pcDNA3.1-PDK1 and pcDNA3.1-PDK2 expression plasmids were bought from Obio Technology (Shanghai, China).

### Western blot

Whole cell lysates were prepared and Western blot was performed as previously described [35]. The antibodies for β-actin and PDHE1α were from Abcam Company (San Francisco, CA, USA), antibodies for Bcl-2 and Mcl-1were from Santa Cruz Biotechnology (Santa Cruz, CA, USA), and the antibodies for PARP, BCL-xL, GSK-3β, p-ERK (Thr^202^/Tyr^204^), p-JNK (Thr^183^/Tyr^185^), p-Mcl-1 (Thr^163^), p-Akt (Ser^473^), p-4EBP1, p-mTOR and p-GSK-3β (Ser^9^) were from Cell Signaling Technology (Boston, MA, USA). The antibody against p-PDHE1α (Ser^293^) was from EMD Millipore (Billerica, MA, USA).

### Cell transfection

SKOV3 and OVCAR3 cells were grown to 60% to 70% confluence in 6-well plates. The siRNA or expression plasmid was mixed with 10 μL lipofectamine 2000 in Opti-MEM (Invitrogen, Carlsbad, CA, USA) for each well according to the manufacturer's protocol. After incubated with the mixtures for 6 h, the cells were cultured in DMEM with 10% FBS for another 6 h. Then the cells were given the corresponding treatment.

### Cell viability assay

Cell viability assay was performed using CCK-8 kit (Dojindo, Shanghai, China) as previously described [27]. Briefly, cells were seeded onto 96-well plates (2×10^3^ cells/well) in triplicate and incubated for 12 h. Then the cells were given different treatment or vehicle control for 48 h, followed by the addition of 10 μl CCK-8 solution to each well. After incubation at 37°C for 1.5 h, the value of OD_450nm_ was determined with a microplate reader.

### Flow cytometry

Ovarian cancer cells were incubated with annexin V-FITC and PI according to the manufacturer's instructions (BD, 561012). Then the apoptosis were analyzed by a flow cytometer.

### Hoechst staining

After treated for 24 h, the cells were stained with Hoechst 33258 (Beyotime, Shanghai, China) at 10μg/mL for 10 min at dark. Subsequently, the cells were washed 3 times with PBS and photographed under afluorescence microscope.

### Caspase 3 activity assay

Caspase 3 activity was examined with Caspase3 Activity Assay kit (Beyotime, C1115) as previously described [36]. Briefly, the control and treated cells were harvested, washed with ice-cold PBS, and resuspended in 50 μl of chilled cell lysis buffer for 15 min on ice. Then the lysates were centrifuged (20,000 g, 10 min, 4°C), and the supernatants were collected for caspase3 activity assay immediately.

### Measurement of total intracellular ROS

The total intracellular ROS was tested with ROS Assay Kit (Beyotime, Shanghai, China) as previously described [37].

### RNA isolation, quantitative real-time PCR (qPCR)

Total RNA was extracted from the cells with TRIzol reagent (ComWin Biotechnology, Beijing, China) and the first-strand cDNA was synthesized using M-MLV transcriptase (Invitrogen, Carlsbad, CA, USA). The qPCR was done with QuantiFast SYBR Green PCR Kit (Promega, Shanghai, China). The relative mRNA levels of the target genes were calculated with 2^−ΔΔCt^ method.

### Analysis of green fluorescent protein (GFP)-MAP1LC3

After transfected with the GFP-MAP1LC3 (GFP-LC3) expression vector for 12h, the cells were given the indicated treatments for another 24 h and then fixed with 4% formaldehyde for 10 min. Subsequently, the cells were washed 3 times with PBS and observed under a fluorescent microscope.

### Detection of L-lactate and glucose

The cells were treated in 6-wells, and then the medium was collected and the concentrations of L-lactate and glucose were determined separately using L-Lactate Assay Kit (Eton Bioscience, San Diego, CA, USA) and Glucose Colorimetric/Fluorometic Assay Kit (BioVision, Milpitas, CA, USA).

### Cellular bioenergetics analysis

The cells were plated in XF96 plates and allowed to grow overnight. Then the media were replaced with XF96 media 1 h before the assay. Rotenone/antimycin A, FCCP, and oligomycin were diluted into XF96 media and loaded into the accompanying cartridge to achieve final concentrations of 0.5μM, 0.5μM, and 1.0μM, respectively. Injections of the drugs into the medium occurred at the time points specified. The OCR (pmol/min) and ECAR (mpH/min) were monitored with the XF Cell Mito Stress Test Kit (Seahorse Bioscience, North Billerica, MA, USA) using Seahorse Bioscience XF^e^ and XF Extracellular Flux Analyzers.

### Animal study

Six-week-old female nude mice were bought from Beijing Huafukang Bioscience (Beijing, China), and housed and cared for under the regulations of the guidelines of the Animal Care and Ethics Committee of Third Military Medical University (Chongqing, China). 5×10^6^ SKOV3 cells in 150 μL PBS were implanted into the right axillae of each nude mouse. When palpable tumors were formed, the mice were randomized into 4 groups (n = 6 per group). Then the mice were intraperitoneally injected everyday with DCA (50 mg/kg/d) plus Met (100 mg/kg/d) or each alone for 8 days, taking PBS as control. The xenograft tumor size was monitored every day with sliding caliper, and the volume was estimated using the following formula: volume = width^2^×length×1/2. After excised from the mice, the xenograft tumors were photographed, and the corresponding proteins were examined by Western blot.

### Statistical analysis

The data were expressed as mean ± SD. One-way ANOVA and *t*-test were used to analyze the variance. *P*< 0.05 was considered as statistical significant.

## SUPPLEMENTARY FIGURES


