# Supplementary material for: Dichloroacetate and metformin synergistically suppress the growth of ovarian cancer cells
Source: Oncotarget. 2016 Jul 19;7(37):59458–70. doi: 10.18632/oncotarget.10694 (PMC5312324; doi:10.18632/oncotarget.10694)
Supplement: Supplementary file 1 [file oncotarget-07-59458-s001.pdf]

Dichloroacetate and metformin synergistically suppress the growth of ovarian cancer cells

SUPPLEMENTARY FIGURES

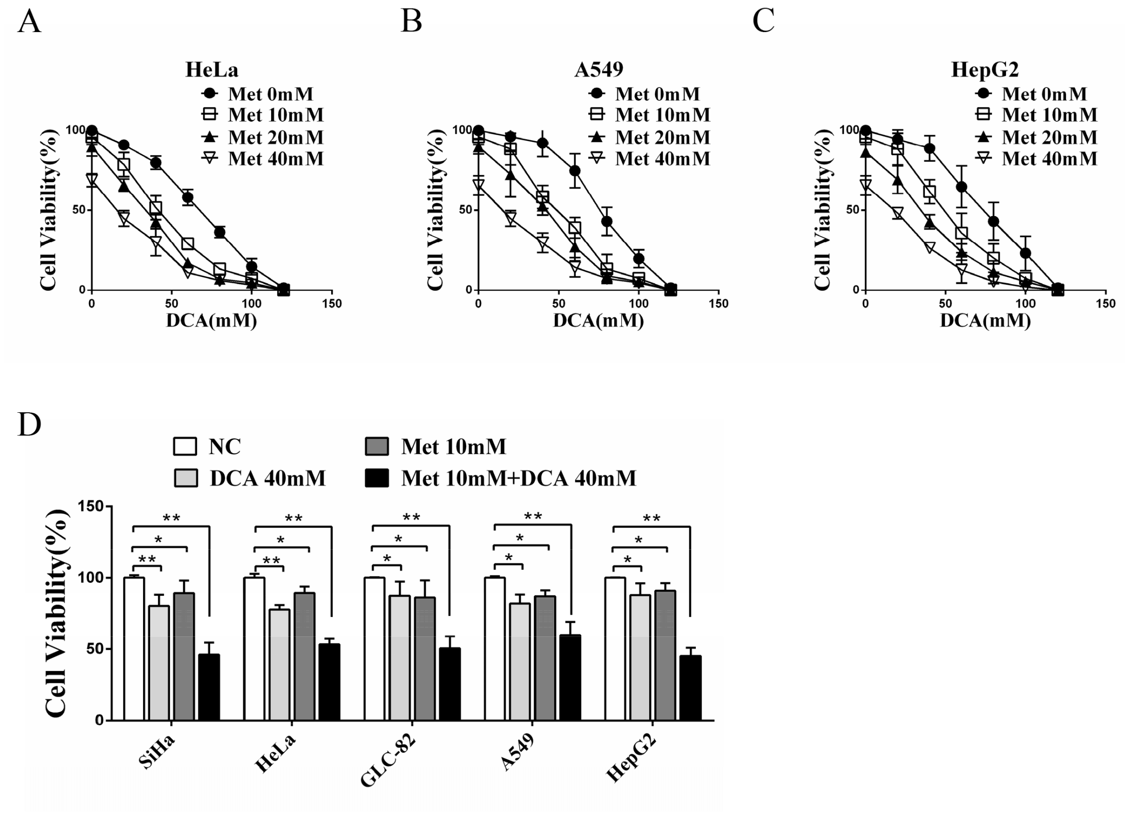

Supplementary Figure S1: DCA and Met synergistically suppress the growth of apoptosis in cervical cancer cells, human lung adenocarcinoma cell lines and human hepatocellular carcinoma cells. A-C. HeLa, A549 and HepG2 cells were cotreated with DCA and Met at the indicated doses for 48 h, and then the cell viability was measured by CCK8 assay. **D.** HeLa, SiHa, GLC-82, A549 and HepG2 cells were cotreated with 40 mM DCA and 10 mM Met or each alone for 48 h, and then the cell viability was measured by CCK8 assay. *,$P<0.05$; **,$P<0.01$.

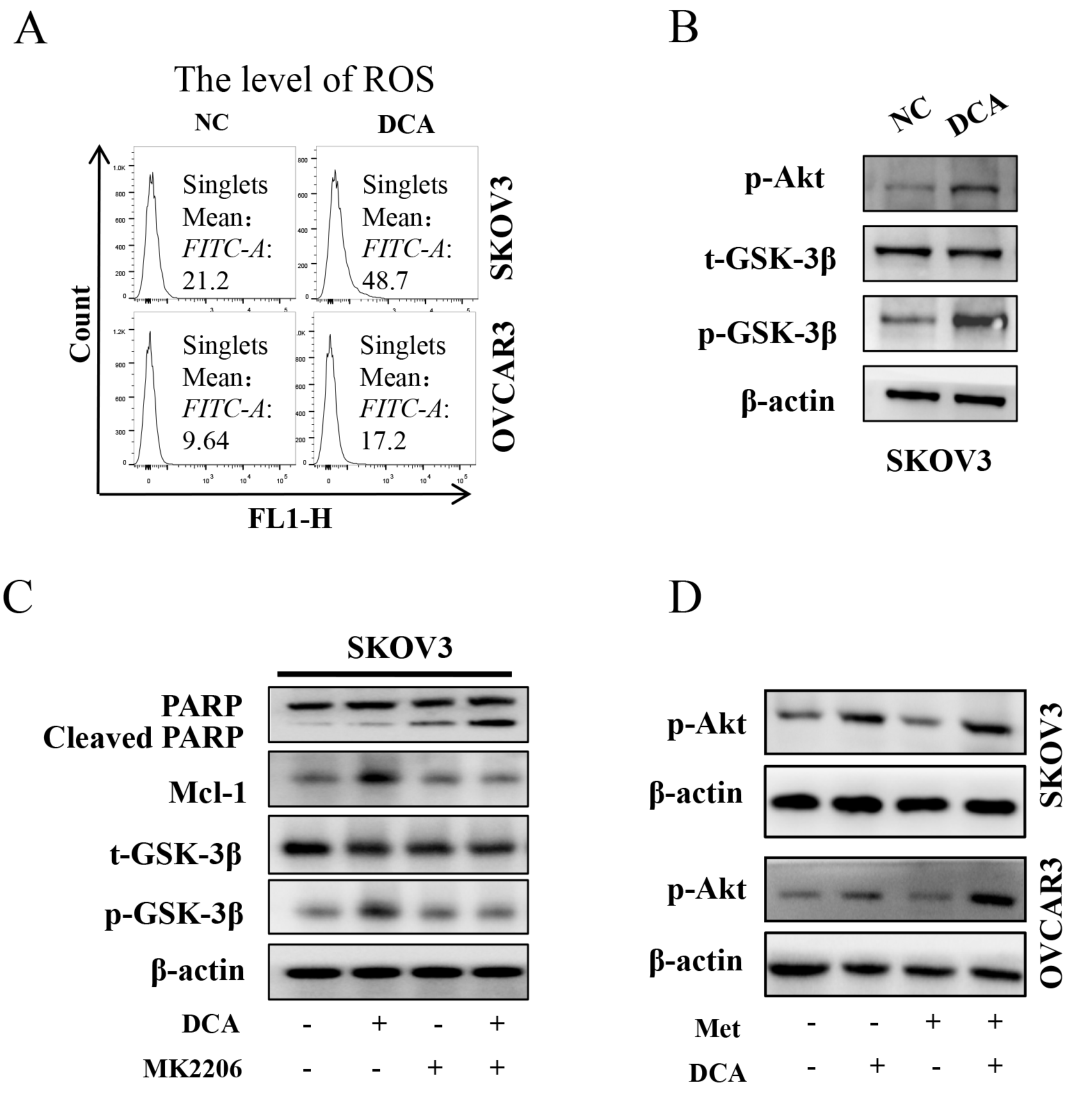

Supplementary Figure S2: The effect of DCA/Met on the production of ROS and levels of p-GSK-3β and p-Akt. A, B. Ovarian cancer cells were treated with 40 mM DCA or PBS control for 24 h, and then the level of ROS was measured with DCFH-DA (A), and p-GSK-3β, p-Akt and total GSK-3β were examined by Western Blot (B). **C.** After pretreated with 5μM MK-2206 (p-Akt inhibitor) or vehicle control DMSO for 2 h, SKOV3 cells were treated with 40 mM DCA for another 24 h. Then the levels of GSK-3β, p-GSK-3β, Mcl-1 and cleaved PARP were analyzed by Western blot. **D.** The cells were cotreated with 40 mM DCA and 10 mM Met or each alone for 24 h, and then the level of p-Akt was examined by Western blot.

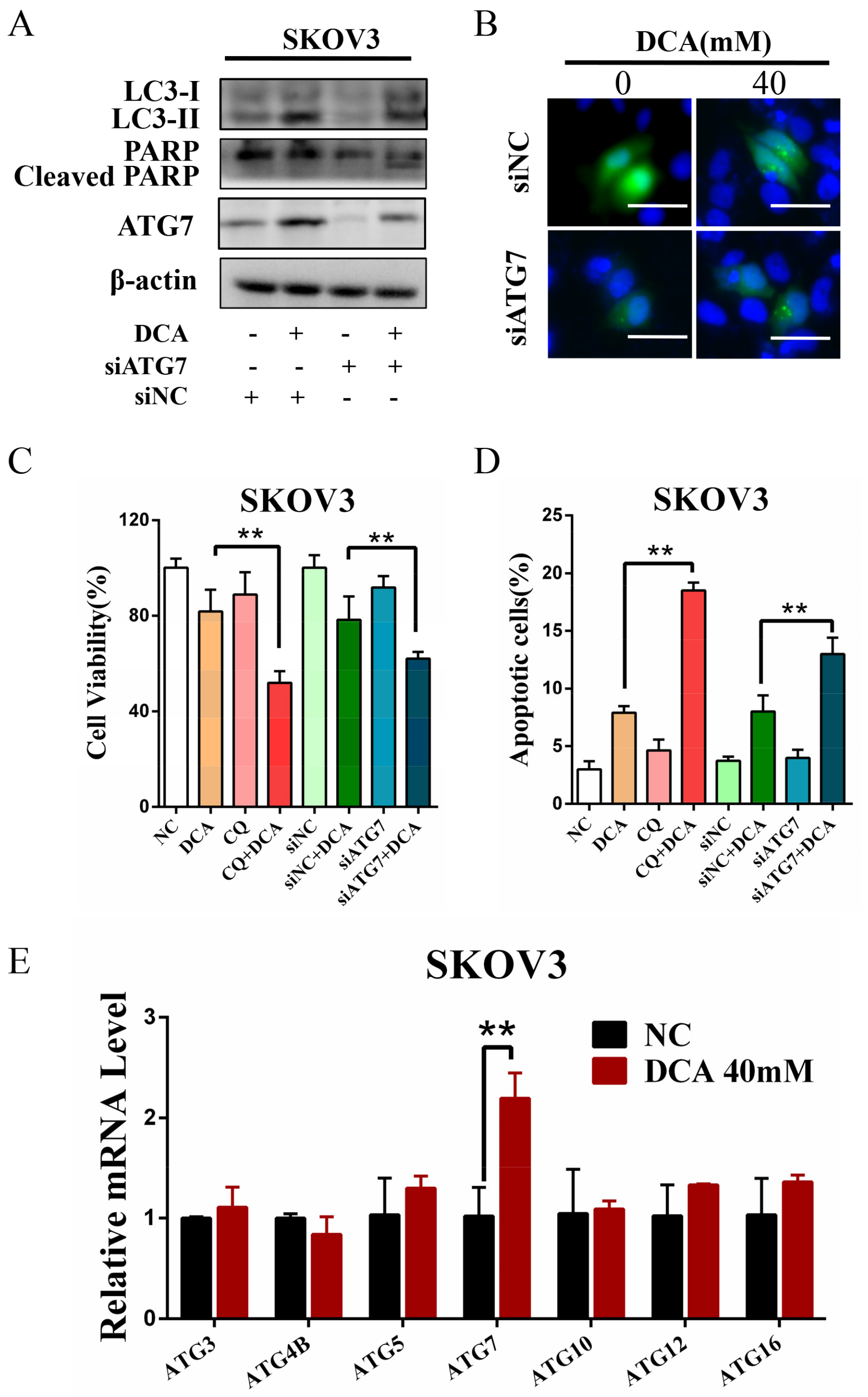

Supplementary Figure S3: DCA induces protective autophagy. A. After trasfected with the siRNA for ATG7 (siATG7) or control siRNA for 12 h, SKOV3 cells were treated with 40 mM DCA for another 24 h. Then the levels of LC3-I/II and cleaved PARP were analyzed by Western blot. **B.** After cotransfected with GFP-LC3 expressing plasmid and siATG7 for 12 h, SKOV3 cells were treated with 40 mM DCA for another 24 h. Then the green fluorescent GFP-LC3 punctas were observed under fluorescent microscope. **C.** After treatment with 40mM DCA in the presence or absence of 20mM chloroquine (CQ) for 24 h, the cell viability was examined using CCK-8 assay. Moreover, the cells were transfected with siATG7 for 12 h, followed by the treatment with 40mM DCA for 24 h. Then the cell viability was detected. **D.** The cells were treated as in (C), and then the apoptotic cells were analyzed using flow cytometry (D). **E.** After treated with 40mM DCA for 24 h, the mRNA levels of 7 autophagy-related genes in the cells were measured by qPCR, and the data was expressed as the fold change over the control. **,*P*<0.01.
